# A. V. M. A. Committee on Diseases

**Published:** 1901-12

**Authors:** E. M. Ranck, James Law, A. T. Peters, L. Frothingham, R. R. Dinwiddie

**Affiliations:** *Chairman*, 21 N. 41st St., Philadelphia.; Ithaca, N.Y.; Lincoln, Neb.; Boston, Mass.; Fayetteville, Ark.


### CORRESPONDENCE.

### A. V. M. A. COMMITTEE ON DISEASES.

EDITOR JOURNAL COMPARATIVE MEDICINE, ETC.:

THE Committee has outlined for its work for the present year the study of "anthrax," to determine accurately the prevalence of the disease, its differential diagnosis, bacteriological investigation, efficiency of and dangers attending vaccination, modes of transmission, post-mortem appearances, and all data pertaining to this violent scourge.

It is the earnest desire of this Committee to have reports from every reader of the JOURNAL, in the hope of adding to the summarized report some phase of the problem and means of combating this disease. The authors of these reports will be given due credit in this report, which will be published in the proceedings of the A. V. M. A. Answers to the following questions will be welcomed by the chairman of this Committee:

1. To what extent is anthrax prevalent in your vicinity?
2. What has been your experience in treating this disease?
3. Have you used anthrax vaccine? If so, what has been your method and what have you accomplished by its use?
4. What other diseases, in your opinion, are similar in general appearances, and how do you make a differential diagnosis?
5. How do you dispose of the carcasses? and give your opinion as to the very best practical method.
6. Have you noticed how long the vaccine protects against the disease, or how long a mild attack renders the host immune against a subsequent one?
7. Have you noticed any differences in the virulence of the disease which could be attributed to climatic conditions?
8. What, in your opinion, is the greatest factor in the transmission of the disease?
9. While making post-mortems did you notice any distinct features or conditions which were contrary to the general text-book description?

The Committee will be pleased to learn of anything relating to this disease which is original with the practising veterinarian or bacteriologist, and any suggestions will be welcomed relating to the various theories, in the hope of establishing a good general outline for the profession to follow in preventing and combating these outbreaks.

E. M. RANCK, *Chairman*, 21 N. 41st St., Philadelphia.
JAMES LAW, Ithaca, N. Y.
A. T. PETERS, Lincoln, Neb.
L. FROTHINGHAM, Boston, Mass.
R. R. DINWIDDIE, Fayetteville, Ark.
*Committee.*

